# Phase protection of Fano-Feshbach resonances

**DOI:** 10.1038/s41467-020-14797-w

**Published:** 2020-02-21

**Authors:** Alexander Blech, Yuval Shagam, Nicolas Hölsch, Prerna Paliwal, Wojciech Skomorowski, John W. Rosenberg, Natan Bibelnik, Oded Heber, Daniel M. Reich, Edvardas Narevicius, Christiane P. Koch

**Affiliations:** 1 Theoretische Physik, Universität Kassel, Heinrich-Plett-Straße 40, 34132 Kassel, Germany; 2 Dahlem Center for Complex Quantum Systems and Fachbereich Physik, Freie Universität Berlin, Arnimallee 14, 14195 Berlin, Germany; 3 Department of Chemical and Biological Physics, Weizmann Institute of Science, Rehovot, 76100 Israel; 4 Department of Particle Physics and Astrophysics, Weizmann Institute of Science, Rehovot, 76100 Israel; 5 Present Address: JILA, NIST and the Department of Physics, University of Colorado, Boulder, CO 80309 USA; 6 Present Address: Laboratorium für Physikalische Chemie, ETH Zürich, 8093 Zürich, Switzerland; 7 Present Address: Department of Chemistry, University of Southern California, Los Angeles, CA 90089 USA

**Keywords:** Excited states, Atomic and molecular collision processes, Quantum mechanics, Imaging techniques

## Abstract

Decay of bound states due to coupling with free particle states is a general phenomenon occurring at energy scales from MeV in nuclear physics to peV in ultracold atomic gases. Such a coupling gives rise to Fano-Feshbach resonances (FFR) that have become key to understanding and controlling interactions—in ultracold atomic gases, but also between quasiparticles, such as microcavity polaritons. Their energy positions were shown to follow quantum chaotic statistics. In contrast, their lifetimes have so far escaped a similarly comprehensive understanding. Here, we show that bound states, despite being resonantly coupled to a scattering state, become protected from decay whenever the relative phase is a multiple of *π*. We observe this phenomenon by measuring lifetimes spanning four orders of magnitude for FFR of spin–orbit excited molecular ions with merged beam and electrostatic trap experiments. Our results provide a blueprint for identifying naturally long-lived states in a decaying quantum system.

## Abstract (web summary)

The lifetime of decaying quantum states has been thought to depend on the strength of the coupling causing the decay. Here the authors demonstrate that quantum mechanical interference can dominate this process, observing Fano-Feshbach resonance lifetimes covering several orders of magnitude.

## Introduction

Fano-Feshbach resonances (FFR) describe decay of quantum mechanical bound states due to coupling with a continuum of scattering states, which may be due to an effective nucleon–nucleon interaction in nuclear physics^1^, configuration interaction in autoionization^2^, spin–orbit interaction in rovibrational predissociation^3^, or hyperfine interaction in ultracold gases^4^. While the energy positions of FFR were shown to follow quantum chaotic statistics^5,6^, an intuitive picture explaining the large range of FFR lifetimes is so far missing. A qualitative understanding of FFR lifetimes is given by the first order perturbation theory, with Fermi’s golden rule predicting them in terms of the coupling strength at resonance. Within this framework, the phenomenon of phase protection readily explains the lifetime variation, requiring just the most basic concepts of quantum theory.

Assuming the bound state to be an oscillator eigenstate weakly coupled to a flat continuum and taking the coupling to be constant, the FFR lifetime is simply given by the overlap of the oscillator state $\left|n\right\rangle$ with the plane wave $\left|k\right\rangle$ that is resonant. This overlap is the Fourier transform ${\tilde{\psi }}_{n}(k)$ of the *n*th oscillator eigenfunction. The lifetime of $\left|n\right\rangle$ becomes infinite if the overlap vanishes (provided there is no further decay mechanism), i.e., at the roots of the Fourier transform. When this picture refers to the radial motion of particles colliding in three-dimensional space, the plane waves are replaced by scattering functions of the form $\sin (kR+\delta )$ with *R* the internuclear separation and *δ* the scattering phase shift. For the sake of simplicity, we take *δ* to be energy independent; and a generalization to partial waves with *l* > 0 is given under the Methods section. Zero overlap with a bound state $\left|n\right\rangle$ then corresponds to the condition of vanishing imaginary part of ${e}^{i\delta }{\tilde{\psi }}_{n}(k)$, or, equivalently,

1 $$\arg \left[{\tilde{\psi }}_{n}\left(k\right)\right]+\delta =m\pi \quad {\rm{with}}\quad m\in {\mathbb{Z}}.$$

**Fig. 1:**
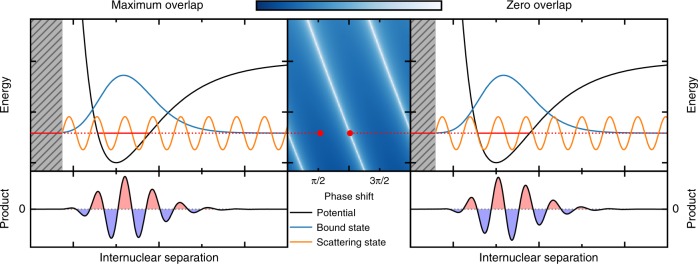
Mechanism of phase protection of a FFR. The overlap of bound and continuum state determines, at least within the perturbative limit, the lifetime of an FFR: vanishing overlap results in infinite lifetime, indicated by white lines in the center panel. The scattering phase is determined by the position of the scattering channel's repulsive wall, here assumed to be a hard wall, as indicated by the gray shaded area.

Equation (1) implies that, for a given scattering momentum *k*, there exist phase shifts *δ* such that the complex overlap between bound state $\left|n\right\rangle$ and scattering state $\left|k,\delta \right\rangle$ vanishes. The lifetime of $\left|n\right\rangle$ may thus become infinite despite non-zero coupling strength. This is illustrated in Fig. 1. While in a typical scattering event, *k* and *δ* cannot be tuned independently, external field control modifies the phase and may thus indeed allow for extremely large lifetimes. To date, two rather different realizations of this phenomenon have been observed, respectively, suggested—magnetic field control of *s*-wave collisions in ultracold atomic gases^7^ and electric dc field control of atomic photoionization^8^. While the required dc field strengths in the latter case are not yet available in experiment, magnetic field control over the lifetime of an FFR in ultracold collisions has been demonstrated experimentally^9^. In that case, the magnetic field modifies the *s*-wave scattering length, which is directly linked to the *s*-wave scattering phase shift^4^.

Here, we show that extremely long FFR lifetimes can also be observed without external field control, just by ensuring that the phase protection condition is fulfilled for certain bound states. Specifically, we consider rovibrational FFR in two different rare gas diatomic ions. We calculate their lifetimes from first principles, connecting peaks in the lifetimes to the phase protection condition. We provide evidence for short-lived FFR in HeAr^+^ and long-lived FFR in NeAr^+^ by velocity map imaging (VMI) the products of merged beam Penning and associative ionization, and corroborate the large range of lifetimes predicted for NeAr^+^, using an electrostatic ion beam trap (EIBT) experiment.

## Results

### Theoretical prediction

**Fig. 2:**
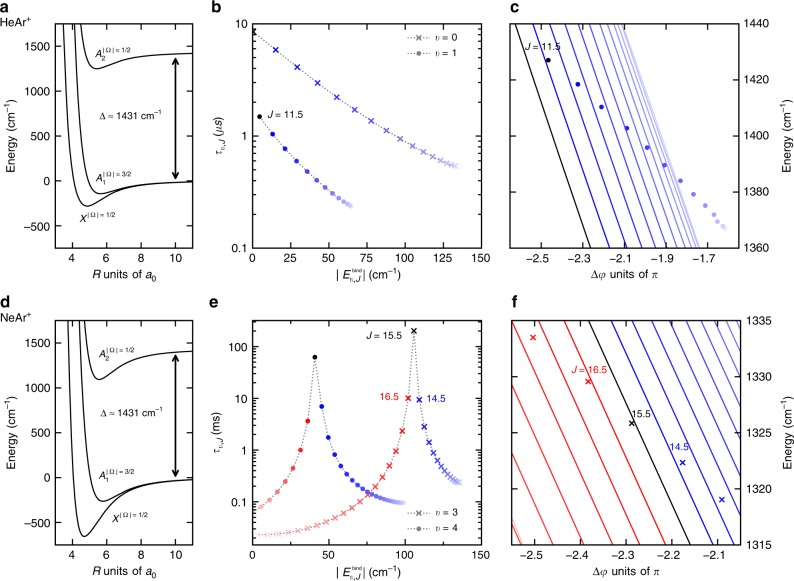
Rovibrational FFR in spin–orbit excited noble gas dimers. Potential energy curves (**a**, **d**) lifetimes of *A*_2_ state rovibrational levels, obtained with full account of non-perturbative effects, as a function of binding energy (**b**, **e**) and zero-overlap lines resulting in phase protection for fixed *v* and different *J*, as a function of scattering phase and energy (**c**, **f**) for HeAr^+^ (**a**–**c**) and NeAr^+^ (**d**–**f**).

Our starting point is the condition for phase protection, Eq. (1). Since $\hslash k=\sqrt{2\mu E}$, the number of roots of ${e}^{i\delta }{\tilde{\psi }}_{n}(k)$ for a given energy increases with the reduced mass *μ*. This is true also when evaluating the phase protection condition with the true scattering states instead of the sine wave approximation. In other words, for a given energy *E*, the chance to fulfill the phase protection condition increases with *μ*. We consider FFR in two different rare gas diatomic ions, HeAr^+^ and NeAr^+^. Rovibrational bound states in the *A*_2_ potential energy curve, cf. Fig. 2a, d, with binding energy ${E}_{v,J}^{{\rm{bind}}}$, have a finite lifetime due to the spin–orbit interaction that couples them to the scattering continuum of the electronic ground state *X*. While the coupling strength is identical for the two molecules, their reduced mass differs by a factor of 3.66, and NeAr^+^ shows stronger binding in all of the relevant electronic states. As described in the Methods section, our first-principles-based theoretical model accounts non-perturbatively for relativistic and angular couplings. Phase protection is observed in the lifetimes’ rotational progression for several vibrational states of NeAr^+^. This is shown in Fig. 2e for two vibrational quantum numbers, which carry significant population in the experiments discussed below. Their peak lifetimes differ from the shortest ones by four orders of magnitude. A similar behavior of lifetimes vs rotational energy has also been observed for HeNe^+^ (ref. ^3^). In contrast, phase protection does not play any role for HeAr^+^, where lifetimes differ at most by a factor of 10 (of the vibrational levels with significant population in the two experiments, the two with the largest lifetime variation have been chosen for display in Fig. 2). Figure 2c, f explains this observation by comparing how far the actual energies and phases of the resonant scattering states (indicated by crosses) are from the lines of vanishing overlap, i.e., from fulfilling the phase protection condition. Note that, for each rotational quantum number *J*, we get a plot as shown in the center of Fig. 1, due to the *J* dependence of the rotational barrier. For ease of comparison, we only show the closest line of vanishing overlap in Fig. 2c, f. We find that the closer a resonance is placed to its zero-overlap line, the larger becomes its lifetime, confirming the expectation from the phase protection condition. The difference between HeAr^+^ and NeAr^+^ is thus understood in terms of sampling in the phase–energy plane. Approximating the *A*_2_ state by a Morse oscillator, cf. the Methods section, we find the number of roots, i.e., the density of zero-overlap lines or the chance of fulfilling the phase protection condition, to increase with reduced mass as well as depth and equilibrium position of the potential and to decrease with potential width. This estimate from the Morse potential approximation is in accordance with the ab initio data for HeAr^+^ and NeAr^+^, cf. Fig. 2, since NeAr^+^ possesses the larger reduced mass and deeper *A*_2_ state potential, whereas width and equilibrium distance are very similar for the two molecules.

### Evidence for long-lived resonances in VMI

**Fig. 3:**
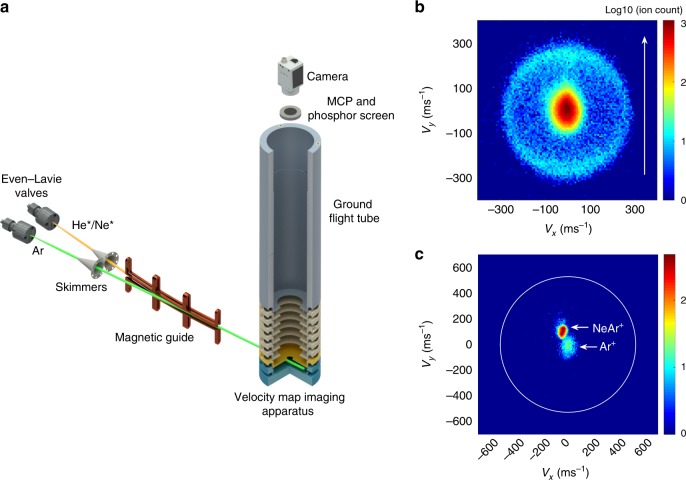
VMI experiment and results. Schematic setup of the VMI experiment (**a**) together with images for Ar^+^ from He*–Ar collisions (**b**) and Ar^+^ and NeAr^+^ from Ne*–Ar collisions (**c**), collected at collision energies *E*∕*k*_B_ = 7.8 K and 14.0 K, respectively. The outer ring observed for Ar^+^ (from He*–Ar) corresponds to a kinetic energy comparable to the spin–orbit splitting, cf. Fig. 2, and indicates presence of FFR with lifetimes shorter than the TOF (~12 μs). The absence of such a ring (the expected position is indicated by the white circle in **c**) for Ar^+^ from Ne*–Ar suggests lifetimes significantly exceeding the TOF. The axes in **b**, **c** correspond to Ar^+^ velocities, where the collision axis lies along the green trajectory in **a** and points up in the VMI images as depicted by the white arrow in **b**, **c**. The image in **b** is taken with temporal mass selection of the Ar^+^ products, while in **c** the different masses are spatially separated on the detector by an external magnetic field as described in the Methods section.

Experimental evidence for the presence of short-lived FFR in HeAr^+^ and long-lived FFR in NeAr^+^, as predicted theoretically in Fig. 2, is provided by VMI of ions resulting from Penning and associative ionization processes occurring in a merged beam apparatus. In these experiments, as sketched in Fig. 3a, neutral argon atoms are ionized upon collision with ^3^*S*_1_ metastable helium or ^3^*P*_2_ metastable neon, respectively. Ionization at large interparticle separation results in atomic products, i.e., Ar^+^ and neutral helium or neon. In contrast, if ionization happens at short interparticle separation, molecular ions are formed^10^ in the three electronic states shown in Fig. 2a, d. Molecules in the *A*_2_ state may decay during the time of flight (TOF), leading to dissociation into Ar^+^, and neutral helium or neon, respectively. The resulting gain in kinetic energy of the ions is of the order of the spin–orbit excitation energy of Ar^+^, which amounts to 1431 cm^−1^. All the ionic products are detected by a VMI setup, cf. the Methods section. The image presented in Fig. 3b shows the velocity distribution of argon ions produced by Penning and associative ionization of argon by metastable helium. The central feature with a width of 35 m s^−1^ is formed by Ar^+^ produced directly by Penning ionization, whereas the ring of 274(±25) m s^−1^ radius is formed by argon ions that are generated by associative ionization into the *A*_2_ spin–orbit excited molecular ion state of HeAr^+^ and subsequent decay and dissociation. According to our calculations, bound levels with *v* = 0, …, 5 with *J* ranging between 0.5 and 15.5 in the *A*_2_ state are populated by the associative ionization. The excess energy, on the order of 1431 cm^−1^, is distributed among the Ar^+^ ion and the neutral helium fragment, leading to a recoil of Ar^+^ on the order of 274(±25) m s^−1^. The Ar^+^ products here are temporally mass selected (cf. the Methods section). The ring is absent when imaging HeAr^+^ separately.

In a striking difference to Fig. 3b, the image shown in Fig. 3c, obtained from the ionization of argon by metastable neon, does not show the outer ring. NeAr^+^ molecular ions are detected in the same image and appear as a focused feature with the same width as the Ar^+^ ions. The molecular ions appear at a different spot due to the deflection by a bias magnetic field applied across the VMI setup, cf. the Methods section. This suggests that the lifetime of spin–orbit excited molecular NeAr^+^ ion significantly exceeds the TOF, which is on the order of several microseconds.

### Lifetime range in EIBT experiment

**Fig. 4:**
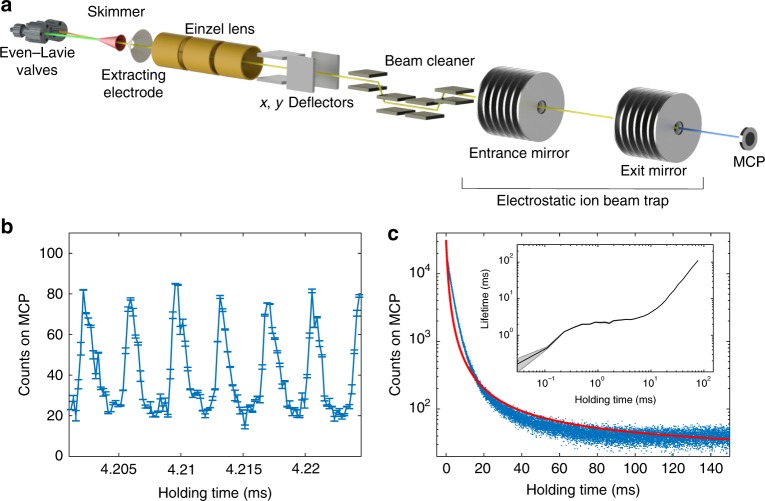
EIBT experiment and results. **a** Experimental setup used to produce and trap a beam of NeAr^+^ and detect the neutral decay products over time. **b**, **c** Neutral product count vs time, after 22,500 injections binned with 2 × 10^−7^ s (**b**) and 1 × 10^−5^ s (**c**). The inset in **c** shows the local lifetime observed during the non-exponential decay with the standard error indicated by the shaded area. The red curve in **c** is the theoretical prediction for the number of molecular ions as a function of time, with the initial populations estimated by an approximate overlap argument as explained in the Methods section.

In order to assess the NeAr^+^ lifetimes and probe, in particular, the predicted lifetime range covering several orders of magnitude, we have carried out a second experiment, cf. Fig. 4, using two crossed molecular beams containing argon and metastable neon to generate molecular ions. These ions were injected into an EIBT^11^ with the apparatus shown in Fig. 4a (see the Methods section for details), where they are trapped for several hundreds of milliseconds, during which they oscillate between two electrostatic mirrors. Collisions with the residual gas as well as predissociation produce neutral particles that leave the trap and are detected by a micro-channel plate detector (MCP). Predissociation of molecules in the spin–orbit excited *A*_2_ state, expected to populate vibrational levels *v* = 3, …, 9 with *J* = 0.5, …, 35.5 given the temperature of the associative ionization reaction, can thus be observed in the decay of the number of trapped ions. Figure 4b shows the counts of neutral particles lost from the trap over time, displaying a distinct oscillation frequency that corresponds to the mass-to-charge ratio of the molecules, matching that of NeAr^+^. Figure 4c depicts the same quantity on a longer time scale together with an inset showing their local lifetimes, obtained by fitting the data to a single exponential in a local segment of the time axis. We find overall non-exponential decay with decay times ranging from <50 μs to 100 ms, in qualitative agreement with the range of predicted lifetimes for the *A*_2_ state shown in Fig. 2e. The agreement with the theoretical prediction of a large range of lifetimes is further corroborated by comparing the experimental data with the red curve in Fig. 4c depicting the sum over all rovibrational level populations as a function of time (see the Methods section for details).

We have shown that quantum resonances may naturally be phase protected without the need for external field control. Occurrence of phase protection depends on the width, depth, and equilibrium position of the bound-state potential and can be modified with reduced mass. Our findings provide guiding principles to identify quantum states that are protected against undesired decay.

## Methods

### Phase protection condition for arbitrary partial waves

Before describing our ab initio model for the molecular structure of the noble gas dimers, we generalize the condition for phase protection, presented in the main text for *s*-waves (*l* = 0), to arbitrary partial waves *l*. Accounting for the rotational barrier, we derive a condition for vanishing radial overlap between a bound state with radial wavefunction *φ*(*R*), that is, *ψ*(*R*) = *R* *φ*(*R*), and a scattering state ${\varphi }_{k,l}(R)=\left\langle R| k,l\right\rangle ={A}_{l}\ {j}_{l}(kR)+{B}_{l}\ {y}_{l}(kR)$, with *A*_*l*_ and *B*_*l*_ real constants and *j*_*l*_ and *y*_*l*_ the spherical Bessel functions of the first and second kind, respectively. The overlap between the bound and scattering state is given by

2 $$\left\langle k,l| \varphi \right\rangle ={A}_{l}{\int }_{0}^{\infty }{R}^{2}\varphi (R)\ {j}_{l}(kR)\ dR+{B}_{l}{\int }_{0}^{\infty }{R}^{2}\varphi (R)\ {y}_{l}(kR)\ dR$$

3 $$=\sqrt{\frac{\pi }{2k}}\left[{A}_{l}{\int }_{0}^{\infty }R\sqrt{R}\ \varphi (R)\ {J}_{l+\frac{1}{2}}(kR)\ dR+{B}_{l}{\int }_{0}^{\infty }R\sqrt{R}\ \varphi (R)\ {Y}_{l+\frac{1}{2}}(kR)\ dR\right]$$

4 $$=\sqrt{\frac{\pi }{2{k}^{2}}}\left[{A}_{l}{H}_{l+\frac{1}{2}}\left[R\ \varphi (R)\right](k)+{B}_{l}{\tilde{H}}_{l+\frac{1}{2}}\left[R\ \varphi (R)\right](k)\right]\ ,$$

where *J*_*n*_ and *Y*_*n*_ are the ordinary Bessel functions of the first and second kind, *H*_*n*_ is the Hankel transform and ${\tilde{H}}_{n}$ the *Y* transform of order *n* (ref. ^12^),

5 $${H}_{n}[f(r)](k)={\int }_{0}^{\infty }f(r){J}_{n}(kr)\sqrt{kr}dr\ ,\quad {\tilde{H}}_{n}[f(r)](k)={\int }_{0}^{\infty }f(r){Y}_{n}(kr)\sqrt{kr}dr\ .$$

Making use of the fact that the coefficients *A*_*l*_ and *B*_*l*_ determine the scattering phase shift, *δ*_*l*_,

6 $$\tan ({\delta }_{l})=-\frac{{B}_{l}}{{A}_{l}}\ ,$$

the condition for vanishing overlap becomes

7 $$\begin{array}{l}\left\langle k,l| \varphi \right\rangle =0 \iff{H}_{l+\frac{1}{2}}\left[R\ \varphi (R)\right](k)+\frac{{B}_{l}}{{A}_{l}}{\tilde{H}}_{l+\frac{1}{2}}\left[R\ \varphi (R)\right](k)=0\\ \qquad\qquad\quad \iff {H}_{l+\frac{1}{2}}\left[R\ \varphi (R)\right](k)=\tan ({\delta }_{l}){\tilde{H}}_{l+\frac{1}{2}}\left[R\ \varphi (R)\right](k).\end{array}$$

For the special case of *s*-wave scattering, the overlap reduces to

8 $$\left\langle k,l=0| \varphi \right\rangle =	\ \frac{1}{k}{\int }_{0}^{\infty }R\ \varphi (R)\left({A}_{0}\sin (kR)-{B}_{0}\cos (kR)\right)dR\\ \sim 	 {\int }_{0}^{\infty }R\ \varphi (R)\ \sin (kR+{\delta }_{0})\ dR\\ =	\ {\rm{Im}}\left[{e}^{i{\delta }_{0}}F\left[R\ \varphi (R)\right](k)\right]$$

where *F* is the Fourier transform defined as usual, $F[f(r)](k)=\int _{-\infty }^{\infty } \, f(r){e}^{ikr}dr$ and *φ*(*R*) is understood to be zero for negative *R*. Setting ${\rm{Im}}\left[{e}^{i{\delta }_{0}}F\left[R\ \varphi (R)\right](k)\right]$ to zero results in Eq. (1) in the main text.

### Model of rovibrational FFR in rare gas diatomic ions

Rovibrational FFR in rare gas diatomic ions arise due to the spin–orbit interaction coupling different non-relativistic electronic states. The Hamiltonian describing the rovibrational motion of a diatomic molecule, including relativistic and angular couplings^13^ can be restricted here to the lowest ^2^Σ^+^ and ^2^Π states and reads

9 $$H={T}_{{\rm{rad}}}+{V}_{{\rm{ad}}}+{H}_{{\rm{SO}}}+{H}_{{\rm{rot}}}$$

with ${T}_{{\rm{rad}}}=-\frac{{\hslash }^{2}}{2\mu }\frac{{d}^{2}}{d{R}^{2}}{\rm{diag}}(1,1,1)$ the radial kinetic energy,

10 $${V}_{{\rm{ad}}}+{H}_{{\rm{SO}}}=\left(\begin{array}{rcl}{V}^{^{2}{\Sigma }^{+}}(R)&-\frac{\sqrt{2}}{3}\Delta &0\\ -\frac{\sqrt{2}}{3}\Delta &{V}^{^{2}\Pi }(R)+\frac{1}{3}\Delta &0\\ 0&0&{V}^{^{2}\Pi }(R)-\frac{1}{3}\Delta \end{array}\right)$$

with *V*^*α*^(*R*) the adiabatic non-relativistic interaction potentials and Δ the spin–orbit interaction, and

11 $${H}_{{\rm{rot}}}=\frac{1}{2\mu {R}^{2}}\left(\begin{array}{rcl}J(J+1)+\frac{9}{4}+\epsilon (J+\frac{1}{2})&\sqrt{2}-\epsilon \sqrt{2}(J+\frac{1}{2})&-\sqrt{2(J(J+1)-\frac{3}{4})}\\ \sqrt{2}-\epsilon \sqrt{2}(J+\frac{1}{2})&J(J+1)+\frac{5}{4}&-\sqrt{J(J+1)-\frac{3}{4}}\\ -\sqrt{2(J(J+1)-\frac{3}{4})}&-\sqrt{J(J+1)-\frac{3}{4}}&J(J+1)-\frac{3}{4}\end{array}\right)$$

with *J* denoting the total molecular angular momentum quantum number, and *ϵ* is a parity index with *ϵ* = ±1 corresponding to *e* and *f* levels, respectively^14^. In Eq. (9), we have neglected the *R* dependence of both the spin–orbit interaction and the angular couplings and taken Δ to be equal to the value of Ar^+^(^2^*P*). This approximation is well justified since for HeAr^+^ these couplings are estimated to differ from their asymptotic values by <1% (ref. ^15^), and a similar behavior is expected for NeAr^+^. We have also neglected the relativistic Cowan–Griffin term^16^ and the diagonal adiabatic correction, both of which are tiny.

While all numerical results are based on Hamiltonian Eq. (9) without further approximations, we have used the Hund’s case (c) representation in our discussion that is appropriate to illuminate the rovibrational structure of HeAr^+^ and NeAr^+^. This implies that *J* and Ω, the body-fixed *z*-projection of total electronic angular momentum, are good quantum numbers, with Ω = 1/2 for states *X* and *A*_2_ (corresponding to ^2^Σ^+^ and ^2^Π_1/2_), and Ω = 3/2 for state *A*_1_ (corresponding to ^2^Π_3/2_).

### Potential energy curves

Empirical adiabatic potential curves obtained by fitting high-resolution spectroscopic data are available^17^ and have been used for the HeAr^+^ ion, whereas ab initio calculations have been carried out for NeAr^+^. The lowest adiabatic ^2^Σ^+^ and ^2^Π electronic states of NeAr^+^ have been obtained from supermolecular calculations employing a spin-unrestricted coupled cluster method with single, double, and noniterative triple excitations [UCCSD(T)], as implemented in the Molpro package^18^. We have used the correlation-consistent augmented aug-cc-pV6Z basis set for both atoms, and an extra set of diffuse even-tempered functions has been added to describe the Ar atom.

### Calculation of lifetimes

Resonance positions and widths have been obtained by adding a complex absorbing potential (CAP) to all three potential energy curves and subsequent diagonalisation of the resulting non-Hermitian Hamiltonian^19^. The calculations were performed on a spatial grid extending from 3 *a*_0_ to 200 *a*_0_, using the Fourier method^20^ with 4095 basis functions. Such a large number of grid points was necessary to properly converge the lifetimes of the narrow NeAr^+^ resonances. The transmission-free CAP of ref. ^21^ with strength ${E}_{\min }=1295\ {{\rm{cm}}}^{-1}$ and absorption range *D* = 20 *a*_0_ was used. With this choice of parameters, the lifetimes are converged with a relative error of ~0.001% for HeAr^+^ and ~0.1% for NeAr^+^. Δ*ϕ* used in Fig. 2c, e refers to the short-range equivalent of the scattering phase shift that is defined asymptotically.

Additionally, the liftimes were calculated perturbatively using Fermi’s golden rule and Hund’s case (c), where the perturbation is given by the radial and rotational couplings. The Hund’s case (c) representation was obtained by a unitary transformation of the full Hund’s case (a) Hamiltonian. The FFR widths are determined as the transition rates of the *A*_2_ rovibrational bound levels to the continua of the *X* and *A*_1_ states. Since there are no radial couplings between the *A*_1_ and *A*_2_ states, the *A*_1_ contribution to the FFR widths, respectively lifetimes, is only minor. Bound and energy-normalized continuum states were obtained by diagonalizing the unperturbed Hamiltonian using the same grid as described above. The transition rates were calculated for the continuum state with energy closest to the energy of the bound level. For NeAr^+^, the perturbative calculations almost exactly reproduce the lifetimes obtained non-perturbatively using the CAP and full couplings. The largest deviations occur at the maximum lifetimes, where the resonance widths become very small and overlap integrals become quite sensitive to the energy and phase of the continuum state. Nevertheless, the positions of the peaks for phase protected states are in full agreement for all rovibrational levels. For HeAr^+^, perturbative and non-perturbative results also agree very well except for *v* = 0 and low *J*, where quantitative deviations up to a factor of four occur. Except for *v* = 0 and odd spectroscopic parity, the lifetimes decrease with *J*. This monotonic dependence is in disagreement with earlier calculations that predicted an oscillatory behavior with rotational excitation^15^. We attribute the disagreement mainly to a different treatment of the couplings between the electronic states involved in the predissociation: Our calculations are based on a coupled channels, fully non-perturbative approach that is more accurate and less prone to numerical instabilities than the adiabatic method employed in ref. ^15^.

### Lifetime scaling with *μ* in Morse oscillator approximation

For both HeAr^+^ and NeAr^+^, the *A*_2_ state interaction potential for *J* = 0 is well approximated around the potential minimum by the Morse oscillator, $V(R)=D\left({e}^{-2a(R-{R}_{0})}-2{e}^{-a(R-{R}_{0})}\right)$ with *D* the well depth, *a* a constant determining the width (with smaller *a* resulting in a wider potential well), and *R*_0_ the equilibrium distance. The decay rates are given by

12 $${\gamma }_{v}\propto \sin {\left(\delta +{\phi }_{v}(k)\right)}^{2}\ {\left|{\tilde{\psi }}_{v}(k)\right|}^{2}\ ,$$

where ${\phi }_{v}(k)=\arg [{\tilde{\psi }}_{v}(k)]$ and ${\tilde{\psi }}_{v}(k)$ the Fourier transform of the Morse oscillator eigenstate. In order to use the standard Fourier transform, we have written $\sin (kx+\delta )=({e}^{ikx}{e}^{i\delta }-{e}^{-ikx}{e}^{-i\delta })/(2i)$. The absolute value squared ${\left|{\tilde{\psi }}_{v}(k)\right|}^{2}$, thus determines the minimum lifetime that can be expected. On top of a smooth variation with *k* (or energy) due to ${\left|{\tilde{\psi }}_{v}(k)\right|}^{2}$, the decay rates *γ*_*v*_ oscillate, with the roots corresponding to phase protection, cf. Eq. (1). Exploiting the fact that ${\tilde{\psi }}_{v}(k)$ is known analytically, in terms of complex Γ-functions^22^, we obtain the scaling of the lifetimes, respectively decay rates, with the parameters of the potential and the reduced mass. Specifically, we find that the number of roots in *γ*_*v*_ and thus the chance for phase protection increase with *D*, *R*_0_, *a*, and *μ*. This agrees well with our observation of phase protection for NeAr^+^ and its absence for HeAr^+^, since the reduced mass and the well depth are larger for NeAr^+^ than for HeAr^+^, while the position of the minimum and the potential width are very similar for the two molecules. More generally, our estimate predicts an isotope effect on predissociation lifetimes, which agrees well with experimental observations for N$$_{2}^{+}\ $$(ref. ^23^) and ${\mathrm{Ne}}_{2}^{+}$ (ref. ^24^).

### Molecular population after associative ionization

The number of molecular ions in the EIBT trap is given by

13 $$P(t)=\left(\sum _{v,J}{p}_{v,J}\ {e}^{-t/{\tau }_{v,J}}+{p}_{{\mathrm{s}}}\right)\ {e}^{-t/{\tau }_{{\mathrm{trap}}}} ,$$

where *p*_*v*,*J*_ is the population of the metastable rovibrational level *v*, *J* in the *A*_2_ state, and *τ*_*v*,*J*_ the corresponding lifetime. The non-dissociating population of rovibrational levels in the *X* and *A*_1_ state is collected in *p*_s_, and *τ*_trap_ is the lifetime due to neutralizing collisions with the background gas in the trap. In order to estimate the populations *p*_*v*,*J*_ after associative ionization, we assume that the rotational angular momentum of the molecule remains unchanged during the ionization, and we model the initial state by a Gaussian wavepacket positioned near the inner classical turning point of the entrance channel potential, with a width corresponding to the spread of collision energies. The populations *p*_*v*,*J*_ are calculated by evaluating the overlap between the wavepacket, multiplied with the optical potential by Baudon et al.^25^, and the rovibrational states of the ion, weighted by the projection of the Gaussian wavepacket onto partial waves. The number of molecular ions in the EIBT trap is directly related to the neutral product count in Fig. 4c (ref. ^11^).

### VMI after merged beam ionization

Cold Penning ionization reactions of He^*^–Ar and Ne^*^–Ar are realized by the merged beam technique described by Henson et al.^26^. The pulsed supersonic beams of He/Ne and Ar are created by two Even-Lavie valves^27^ positioned at a ten degree angle. The He/Ne beam is excited to a metastable paramagnetic state by dielectric barrier discharge (DBD)^28^ and are deflected by a curved magnetic guide such that it is co-propagating with the Ar beam. The short opening duration of the Even-Lavie valves allow collision energies as low as 10 mK × *k*_B_ to be reached^29^.

The two beams meet in the reaction region inside the VMI detector. The beams are skimmed by two parallel razors before the VMI detector to limit their distribution to a 1 mm plane. The VMI setup consists of eight separate plates electrodes positioned perpendicular to the beam path, followed by a 9th grounded electrode. These are followed by a flight tube ~1 m long. The electrode immediately above the beam (second plate) has an aperture of 1 cm in diameter for the ions to pass through, limiting the overall region from which the ions can be imaged to a 1 mm by 10 mm diameter cylinder. The remaining ring electrodes have an aperture of 40 mm diameter.

Argon is ionized upon collision with He(^3^*S*_1_)/Ne(^3^*P*_2_), leading to Ar^+^ as well as HeAr^+^/NeAr^+^ for associative ionization. The ions are then accelerated toward the MCP detector. The MCP is followed by a phosphor screen that facilitates the imaging of the product ions by a charge couple device camera. The center of the individual product ions is found as these are accumulated.

Since the time of ionization is not precisely known for our reaction, we must adjust the VMI method to allow for mass selectivity. To this end, we have used two VMI operation modes: (i) constant voltages mode where particles with different mass-to-charge ratio are spatially separated by an external magnetic field and (ii) a pulsed mode^30,31^ that selects particles with a certain mass-to-charge ratio by TOF. In our experiments, the products from Penning (Ar^+^) and associative ionization (HeAr^+^ and NeAr^+^) for the two reactions were separated using these methods.

(i) Constant voltages with bias magnetic field: in this mode, all eight plates of VMI have constant voltages throughout the measurement. By applying a uniform magnetic field parallel to the neutral beam collision axis, the product ions get shifted on the detector according to their respective mass-to-charge ratios. The VMI resolution is not affected since the magnetic field is uniform. Figure 3c is acquired with a magnetic field of ~10 Gauss along the entire VMI.
(ii) Pulsed mode: this approach combines the idea of velocity mapping with TOF mass spectrometry allowing the imaging of a single mass/charge on the detector. In this method, all the plates are simultaneously pulsed to their respective imaging voltages during the interaction time. The imaging pulse to the VMI electrodes follows a cleanup pulse with a period defining the product accumulation time. To keep the VMI resolution optimal, this timing is limited by the expansion of the products outside the imaging volume. When the imaging pulse is applied, all the ions formed during accumulation time fly up to the detector and get separated during their flight times, allowing TOF mass spectrometry to be performed. The MCP at the end of the flight tube is pulsed on for 50 ns selecting a single mass product for imaging. This method is used for collecting the data for Fig. 3b, which shows Ar^+^ obtained from He*–Ar collisions. Note that the actual size of the image formed on the detector is 5 mm in diameter.

The velocity detected by the VMI is calibrated by creating a cold beam of Rydberg argon atoms at a range of velocities by DBD on a beam of argon. The beam is then field ionized when the VMI is pulsed and its position is detected for every velocity. The velocity of the argon beam is measured by another MCP positioned in front of the argon valve. Both the UV light from the discharge and the metastable argon atoms are detected by the MCP giving the TOF.

### Measurement of decay rates

NeAr^+^ ions were formed in crossed molecular beams of argon and neon, produced by Even-Lavie valves. The neon beam was excited to the metastable ^3^*P*_2_ level with a DBD, with all ions generated during the excitation deflected away. The beams crossed at 12 degrees, their angles respective to the axis of the skimmer. Assuming translational beam temperatures of 1 K, we obtain a collision energy of ~16 meV, which corresponds to a reaction temperature of 190 K with a spread of 10–15 K. The collision products were skimmed before being accelerated to 4.2 keV, directed through several beam manipulation elements and injected into the EIBT trap. The setup is depicted in Fig. 4a, for further details we refer to ref. ^32^. Mass-sensitive detection of the trapped particles by a pick-up electrode was used to optimize the setup parameters such as manipulation voltages and trap timing. Neutral products stemming from dissociation of the ions or neutralizing collisions with the background gas are lost from the trap and were detected on a MCP located on-axis behind the trap. The kinetic energy of Ne arising from dissociation of NeAr^+^ is 2.8 keV, which is enough to be detected on the MCP. NeAr^+^ could be trapped with minimal contaminating ions, as confirmed by FFT analysis of the pick-up signal and the distinct oscillation observed in the neutral count, as depicted in Fig. 4b. The experimental data is binned in 200 ns intervals to bring out the oscillations on the scale of the NeAr^+^ frequency in the trap (Fig. 4b), and in 10 μs intervals for showing the overall decay in Fig. 4c. The local lifetimes are obtained by splitting the time axis into segments such that each data point contributes to at least two adjacent segments. The data within each segment is fit to a single-exponential decay.

Figure 4c shows the data <150 ms, however, the experimental data continues up to 500 ms. The lifetimes range from <50 μs to 100 ms as estimated from the data, cf. the inset of Fig. 4c, and show qualitative agreement with the expected lifetimes indicated in Fig. 2. The lower limit of 50 μs is assessed through a biexponential decay as shown in the Supplementary Fig. 1. The lifetimes of other vibrational states (*v* = 5–9) are theoretically predicted between 0.1 ms and 1 ms. The typical lifetime of the EIBT trap due to background collisions is longer than 1.5 s.

## Supplementary information

Supplementary Information

## Data Availability

All relevant data are available from the authors upon request.
