# Supplementary material for: Phase protection of Fano-Feshbach resonances
Source: Nat Commun. 2020 Feb 21;11:999. doi: 10.1038/s41467-020-14797-w (PMC7035365; doi:10.1038/s41467-020-14797-w)
Supplement: Supplementary file 1 — Supplementary Information [file 41467_2020_14797_MOESM1_ESM.pdf]

# Supplementary Information: Phase protection of Fano-Feshbach resonances

Alexander Blech et al.

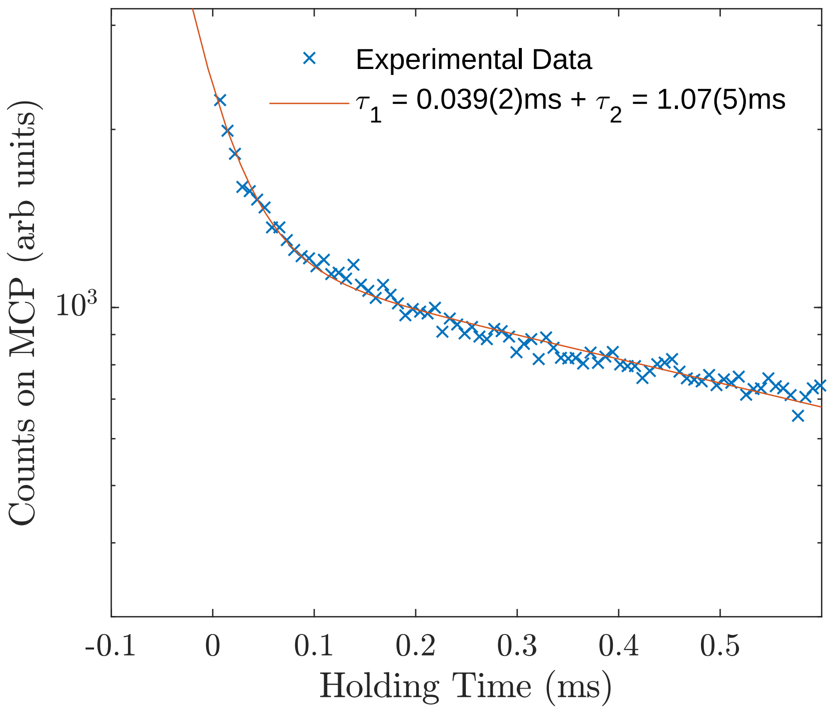


Supplementary Figure 1: Closeup of early times of the decay of $NeAr^+$ molecules in the trap in one of our data sets collected as described in Fig. 3c of the main text. The data is fit to a bi-exponential decay function with lifetimes $\tau_1$ and $\tau_2$.
